# Intermittent body composition analysis as monitoring tool for muscle wasting in critically ill COVID-19 patients

**DOI:** 10.1186/s13613-023-01162-5

**Published:** 2023-07-08

**Authors:** Johannes Kolck, Zvonimir A. Rako, Nick L. Beetz, Timo A. Auer, Laura K. Segger, Christian Pille, Tobias Penzkofer, Uli Fehrenbach, Dominik Geisel

**Affiliations:** 1 Department of Radiology, Charité - Universitätsmedizin Berlin, Berlin, Germany; 2 Department of Pneumology and Intensive Care, Universities of Giessen and Giessen Lung Center (UGMLC), Member of the German Center for Lung Research (DZL), Berlin, Germany; 3 Berlin Institute of Health at Charité - Universitätsmedizin Berlin, Berlin, Germany; 4 Department of Anesthesiology and Intensive Care Medicine | CCM | CVK, Charité - Universitätsmedizin Berlin, Berlin, Germany; 5 Department of Radiology, Charité - Universitätsmedizin Berlin, Corporate Member of Freie Universität Berlin and Humboldt-Universität zu Berlin, Augustenburger Platz 1, 13353 Berlin, Germany

**Keywords:** Critical care, COVID-19, Muscle wasting, Artificial intelligence, Body composition analysis, Computed tomography

## Abstract

**Objectives:**

SARS-CoV-2 virus infection can lead to acute respiratory distress syndrome (ARDS), which can be complicated by severe muscle wasting. Until now, data on muscle loss of critically ill COVID-19 patients are limited, while computed tomography (CT) scans for clinical follow-up are available. We sought to investigate the parameters of muscle wasting in these patients by being the first to test the clinical application of body composition analysis (BCA) as an intermittent monitoring tool.

**Materials:**

BCA was conducted on 54 patients, with a minimum of three measurements taken during hospitalization, totaling 239 assessments. Changes in psoas- (PMA) and total abdominal muscle area (TAMA) were assessed by linear mixed model analysis. PMA was calculated as relative muscle loss per day for the entire monitoring period, as well as for the interval between each consecutive scan. Cox regression was applied to analyze associations with survival. Receiver operating characteristic (ROC) analysis and Youden index were used to define a decay cut-off.

**Results:**

Intermittent BCA revealed significantly higher long-term PMA loss rates of 2.62% (vs. 1.16%, *p* < 0.001) and maximum muscle decay of 5.48% (vs. 3.66%, *p* = 0.039) per day in non-survivors. The first available decay rate did not significantly differ between survival groups but showed significant associations with survival in Cox regression (*p* = 0.011). In ROC analysis, PMA loss averaged over the stay had the greatest discriminatory power (AUC = 0.777) for survival. A long-term PMA decline per day of 1.84% was defined as a threshold; muscle loss beyond this cut-off proved to be a significant BCA-derived predictor of mortality.

**Conclusion:**

Muscle wasting in critically ill COVID-19 patients is severe and correlates with survival. Intermittent BCA derived from clinically indicated CT scans proved to be a valuable monitoring tool, which allows identification of individuals at risk for adverse outcomes and has great potential to support critical care decision-making.

## Introduction

SARS-CoV-2 virus infection can lead to hypoxemic respiratory failure and acute respiratory distress syndrome (ARDS). ARDS is often complicated by intensive care unit (ICU)-acquired weakness (ICUAW), which is marked by severe muscle wasting [1, 2]. Severe muscle loss is common in critically ill patients and usually appears within the first days after admission to the ICU, progressing thereafter [3, 4]. The degree of muscle loss correlates with the severity of the underlying condition and is especially high in patients with sepsis [4]. Patients with a reduced muscle mass at the time of admission have a higher risk of complications, such as prolonged weaning from invasive mechanical ventilation (IMV), longer ICU stay, and a higher mortality [5]. Commonly used methods for estimating and monitoring muscle mass include bioelectrical impedance analysis (BIA) and ultrasound (US). Monitoring by image segmentation tools, offering precise quantification of patient’s tissue components—skeletal muscle area (SMA) and psoas muscle area (PMA), but also subcutaneous adipose tissue (SAT) and visceral adipose tissue (VAT), has been considered previously, but is not yet routinely performed [6, 7]. The increase of imaging data, due to the recommendation of international guidelines to deploy CT scans for the (repeated) assessment of COVID-19 patients [8], has made muscle monitoring using image segmentation tools a viable option for this patient population.

Objectives of the present study were to investigate the parameters of muscle atrophy in critically ill patients suffering from severe ARDS due to SARS-CoV-2 infection while evaluating the clinical applicability of body composition monitoring by means of image segmentation.

## Materials and methods

### Study design

In this cohort study, we retrospectively analyzed body composition metrics in critically ill patients suffering from ARDS due to SARS-CoV-2 virus infection. The study was approved by the Institutional Review Board (Internal registration number: EA4/152/20) and conducted according to the principles of the Declaration of Helsinki.

### Patient population

We searched our database retrospectively for adult patients (> 18 years) treated in one of our university hospital’s ICUs for ARDS due to SARS-CoV-2 infection between March 2020 and January 2022. Inclusion criteria were a minimum of 10 days on invasive mechanical ventilation (IMV), at least 10 days of ICU stay, and availability of at least three serial CT datasets including the abdomen during hospitalization. Of the 112 patients admitted to the designated intensive care units at our institution during the defined period, 26 patients were excluded as they did either not meet the clinical inclusion criteria (ICU and ventilator days). Another 31 patients underwent only 2 or fewer CT examinations relevant to BCA. Among the 54 patients who met the clinical criteria, a total of 239 CT examinations were performed. Patient consent was waived by the ethics committee.

### Body composition analysis

**Fig. 1:**
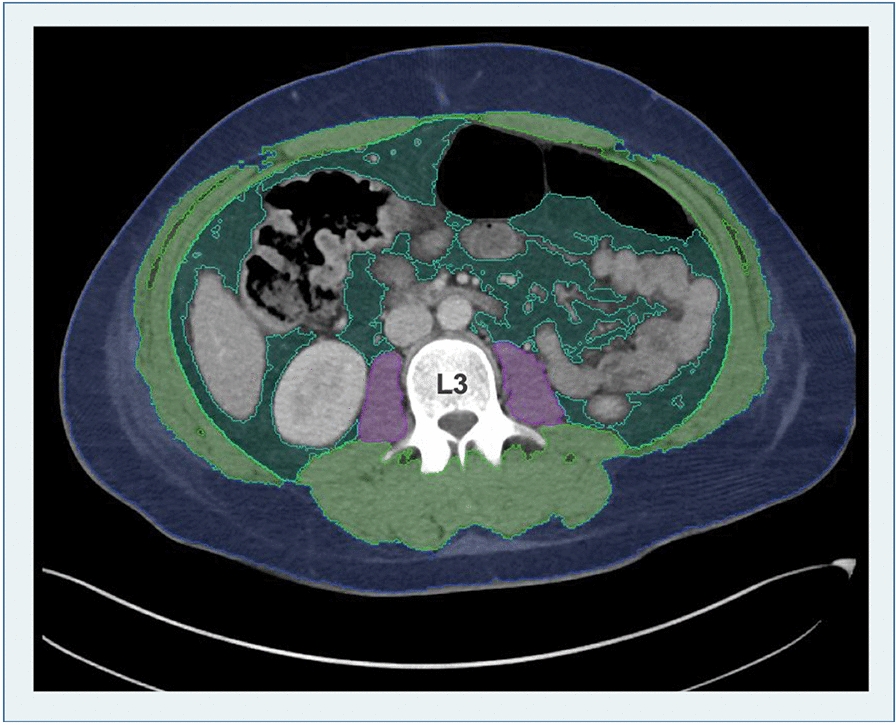
Example of AI-derived, automated body segmentation at the level of lumbar vertebra 3. Segmented tissues are coded with different colors: psoas muscle = purple, skeletal muscle (except psoas muscle) = green, visceral fat = dark green, blue = subcutaneous fat

Body composition was analyzed by applying an AI-based automated image segmentation tool, which is integrated into our Picture Archiving and Communication System (PACS) software (Visage version 7.1., Visage Imaging GmbH, Berlin, Germany) and has been validated in and been used as gold standard in previous studies [7, 9–11]. Following automated identification of the third lumbar vertebra (L3) level, automated segmentation was performed to differentiate tissues into subcutaneous fat (SAT), skeletal muscle area (SMA), visceral fat (VAT), and psoas muscle area (PMA). The software then calculated areas in square centimeters (cm^2^) for each of the four components (Fig. 1). Total abdominal muscle area (TAMA) was calculated as follows: SMA + PMA. Each automated segmentation was checked by an experienced radiologist and manually corrected if necessary.

### Statistics

Descriptive statistics for all numeric variables were calculated as mean and interquartile range (IQR). Analysis of variance (ANOVA) was employed to compare patient-specific differences between survival and non-survival groups, as well as between COVID waves. The COVID waves were defined according to the information provided by the Robert Koch Institute: Wave 1 between 03/2020 to 08/2020, Wave 2 between 08/2020 to 03/2021, and Wave 3 between 03/2021 to 07/2021 [12]. For patients transferred during their illness, we obtained information on the initial hospital admission and, if applicable, admission to the ICU from the transfer letters. In the statistical analysis, we handled hospital admission and ICU admission for transferred patients in the same manner as for patients initially admitted to our institution.

Linear mixed model analysis was employed to examine the repeated measurements of body composition, offering the advantage of accommodating unevenly spaced time points. Relative muscle loss per time point (CT), muscle distribution for age, sex, ECMO therapy, and survival groups were all analyzed independently. Absolute muscle loss per day was calculated for the entire hospital stay (first to last CT) as difference between recorded values for psoas muscle area (in cm^2^) of the first and last CT examination divided by the intervening time interval (in days). Relative muscle decline per day was calculated by dividing the absolute psoas muscle loss per day by baseline PMA of the first available scan (in cm^2^). In the same fashion, the maximum muscle loss between two consecutive CT scans and the loss between the first two scans were calculated. A backward stepwise elimination procedure was utilized to select the most relevant variables for Cox regression from an initial set that included TAMA, VAT Area, SAT Area, PMA, age, BMI, gender, comorbidities, initial ventilation parameters and, the time interval of admission to intubation, as well as above-mentioned first observed PMA loss per day. Non-significant variables were sequentially eliminated. Cox regression was applied to examine the independent associations between various variables and survival. Receiver operating characteristic (ROC) analysis was performed to determine the sensitivity and specificity of relative psoas muscle loss (first, maximum and overall) for survival prediction. The Youden index was calculated to define a potential cut-off for the overall psoas muscle loss per day. Kaplan–Meier curves were calculated for patients above and below the defined cut-off. Statistical analysis was performed with Stata/MP version 16 (StataCorp, College Station, Texas, USA) and SPSS Statistics 27 (IBM, Armonk, NY, USA). All *p*-values < 0.05 were considered statistically significant.

## Results

### Demographics data and preconditions

A total of 54 critically ill patients (38 men and 16 women) with severe ARDS due to SARS-CoV-2 infection who underwent IMV and were admitted to the ICU ward for at least 10 days were retrospectively enrolled. Mean age of the total study population on hospital admission was 55.74 (IQR 48.5–64.25) years. The overall survival rate was 56.6% (30/54 patients). The group of survivors consisted of 10 women and 20 men. Six female and 18 male patients died. Twenty-seven patients suffered from one or more chronic diseases prior to SARS-CoV-2 infection. Arterial hypertension was the most common precondition (24/54 patients), followed by other cardiovascular (10/54 patients), metabolic (8/54 patients), pulmonary (7/54 patients) and malignant conditions (4/54 patients).

### Hospitalization and treatment

All enrolled patients developed severe primary pulmonary ARDS during the course of their disease, as per the criteria defined by the Berlin definition [13]. On average, patients were intubated 4 days after admission and underwent invasive mechanical ventilation (IMV) for 56 days (IQR 31.25–69). Patients admitted to our hospital exhibited a mean oxygenation index of 73.44 (IQR 65.75–77.75) prior to intubation. These patients underwent mechanical ventilation with an initial set positive end-expiratory pressure (sPEEP) of 16.46 (IQR 13.75–18) cmH_2_O, a peak inspiratory pressure (pPeak) of 32 (IQR 29.75–34) cmH_2_O, a respiratory minute volume (RMV) l/min of 8.39 (IQR 7.30–9.60), and a respiratory rate (RR) of 20.20 (IQR 16.75–23.25). Twenty-one patients were transferred to our institution following intubation at an external hospital. Upon transfer, these patients were receiving ventilation with a sPEEP of 16.76.2 (IQR 14–19) cmH_2_O, a pPeak of 31.52 (IQR 29–34) cmH_2_O, a RMV of 8.12 (IQR 6.6–9.80) l/min, and a RR of 19.33 (IQR 18–20). Twelve patients had undergone external initiation of extracorporeal membrane oxygenation (ECMO) therapy. After transfer, the mean initial ventilation was performed with an sPEEP of 12.67 (IQR 12–15) cmH_2_O, pPeak of 24.67 (IQR 22.75–27.5) cmH_2_O, a RMV of 3.51 (IQR 2.53–4.45) l/min, and RR of 15.00 (IQR 12.75–16). Over the entire course, a total of 43 (79.6%) of patients underwent tracheostomy and 37 (68.5%) patients received ECMO therapy, of the latter 48.65% survived (18/37 patients). The predominant type of ECMO applied was veno-venous. Transient veno-arterial ECMO therapy employed in only three cases. The majority of patients (92.6%) underwent prone positioning during their hospitalization. Dexamethasone was administered to 77.36% of patients, tocilizumab to 20.37%, NO therapy to 51.9%, neuromuscular blockers to 57.41%, and dialysis was performed in 81.48% of patients. ANOVA analysis did not reveal any significant differences between survival and non-survival group regarding all above variables. Mean length of in-hospital stay was 80.2 days with an average of 65.17 days in the ICU. Mean length of hospital and ICU stay were significantly shorter in patients who died with 46.13 and 40.88 days, versus 107.5 and 84.60 days in survivors (*p* < 0.001; *p* < 0.001). All patients received parenteral nutrition at least partially during their hospitalization. Parenteral nutrition was started with a gradual increase in the flow rate of the nutritional solution. After reaching the target flow rate, we recorded the intake over 7 days, accounting on average to 1815 (IQR 1431–2150) kcal per day. Average protein intake was 112.43 (IQR 86.40–126.72) g per day. There were no significant differences between survival groups in regard to nutrition. The clinical characteristics of study patients suffering from severe ARDS due to SARS-CoV-2 infection are summarized in Table 1.

**Table 1:** Clinical characteristics of critically ill patient groups with severe ARDS

	*N*	Mean/percent of patients	Quartiles	
			1	3
Age		55.74	48.50	64.25
BMI		29.74	25.10	32.65
Female gender	16	29.63%		
Survival		55.56%		
Hospitalization (days)		80.24	39.50	103.25
ICU (days)		65.17	33.00	83.25
Ventilation days		55.98	31.25	69.00
Intubation in domo	21	38.89%		
sPEEP (cmH_2_O)		16.46	13.75	18.00
pPeak (cmH_2_O)		32.00	29.75	34.00
RMV (l/min)		8.39	7.30	9.60
RR		20.20	16.75	23.25
PaO_2_/FiO_2_		73.44	65.75	77.75
Intubation ex domo	21	38.89%		
sPEEP (cmH_2_O)		16.76	14.00	19.00
pPeak (cmH_2_O)		31.52	29.00	34.00
RMV (l/min)		8.12	6.60	9.80
RR		19.33	18.00	20.00
ECMO ex domo	12	22.22%		
sPEEP (cmH_2_O)		12.67	12.00	15.00
pPeak (cmH_2_O)		24.67	22.75	27.50
RMV (l/min)		3.51	2.53	4.45
RR		15.00	12.75	16.00
ECMO type				
All	37	68.5%		
VV	34	63.0%		
VA	3	5.6%		
Therapy				
Tracheostomy	43	79.6%		
Prone position	50	92.6%		
NO	28	51.9%		
Dexamethasone	42	77.36%		
Tocilizumab	11	20.37%		
NMBA	31	57.41%		
Dialyse	44	81.48%		
Nutrition				
kcal/day		1815.09	1431.11	2150.40
Protein/day (g)		112.43	86.40	126.72

### Patient transfer and image acquisition

The majority of 41 patients were initially admitted to one of 25 external hospitals in the Berlin–Brandenburg area. Patients were transferred to our institution after an average of 8.8 days. On admission to our hospital, we received externally acquired CT studies for 20 of the patients. An average of 4.4 CT scans relevant for body composition analysis were performed per patient. The first CT scan including the abdomen was obtained an average of 11 days after initial hospital admission.

### Initial analysis of body composition

On average, all AI metrics derived from the initial scans were lower in the deceased patient group (D) than in the survivor group (S). However, there were no statistically significant differences between the two groups for mean TAMA (S: 138 (IQR 114.3–157.9) cm^2^; D: 125 [IQR 98.3–151.5]) cm^2^), VAT (S: 190 [IQR 133.6–257.5] cm^2^; D: 180 [126.7–251.0] cm^2^), SAT (S: 270 [160.27–349.8] cm^2^; D: 236 [174.9–262.6] cm^2^), and PMA (S: 14 [11.5–17.0] cm^2^; D: 13 [9.1–15.1] cm^2^).

### Linear mixed model analysis

Repeated-measures analysis using linear mixed model comparison demonstrated a significant average loss per timepoint of 5.4 (CI 4.2–6.7) cm^2^ and 1.3 (CI 1.11–1.53) cm^2^ for skeletal (*p* < 0.001) and psoas muscle area (*p* < 0.001), respectively. Averaged over all assessments, the annual age-related difference in PMA was 0.66 (CI 0.01–1.38) cm^2^, for TAMA it was 0.17 (CI 0.22–0.56) cm^2^. Significant overall differences of PMA and TAMA were observed between gender groups (*p* = 0.005 and *p* < 0.001): on average, PMA was 2.64 (CI 0.78–4.49) cm^2^ and TAMA 19.58 (CI 9.72–29.44) cm^2^ smaller in women than in men. PMA and TAMA also differed significantly (*p* = 0.012 and *p* < 0.001) between patients who received ECMO therapy and those who did not. The latter group had on average 2.47 (CI 0.55–4.39) cm^2^ greater PMA and 20.37 (CI 10.15–30.60) cm^2^ greater TAMA.

### Muscle loss in the course of hospitalization

**Fig. 2:**
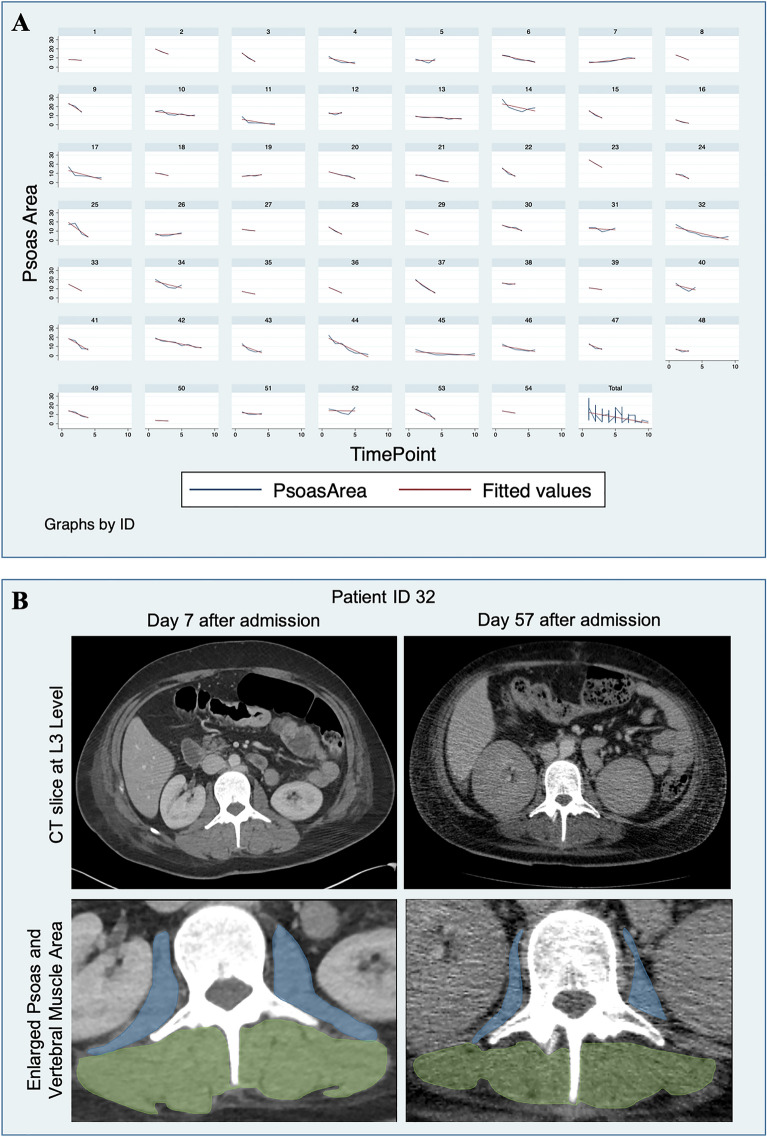
**A** Intermittent monitoring of psoas muscle area in 54 critically ill patients with ARDS due to SARS-CoV-2 infection. Metrics were collected at at least three time points using an AI-based segmentation tool applied to routinely performed CT scans. **B** Depicted are two CT slices used to perform body composition analysis at the level of lumbar vertebra 3 and the respective magnifications for better visualization of the psoas muscle. These example measurements, taken on days 7 and 57 after hospital admission, are from a young patient (ID 32) who survived the infection. Within the 50 days separating the scans, there is a distinct decrease in area of the psoas (blue) and the autochthonous back muscles (green)

In our study, measurement of PMA proved to be least susceptible to fluid accumulation in soft tissues. Mean relative PMA loss between the first and last CT scan was 1.88 (IQR 0.07–2.33) % per day. In the survivor group, average absolute loss in PMA per day was 0.17 (IQR 0.06–0.27) cm^2^/day (*n* = 25) versus 0.36 (IQR 0.17–0.43) cm^2^/d in non-survivors (*n* = 24). Relative PMA decay per day was 2.62 (IQR 1.4–3.0) % in non-survivors and 1.16 (IQR 0.5–1.8) % in survivors. Both relative and absolute muscle decline diverged statistically significantly between the two groups (*p* < 0.001; *p* < 0.001). In more detail, the observed muscle loss demonstrated a non-linear trajectory, characterized by varying rates of decline at different time points, yet exhibiting an overall negative trend in most cases. In 48% of patients the main loss occurred between the first two available CT scans, obtained after 22.76 (IQR 14.0–32.0) days, accounting for 2.82 (1.88–3.76) % PMA loss per day. In 52% the main loss occurred at a later timepoint, in average after 31 (IQR 25.75–36.5) days, amounting for 4.47 (3.52–5.42) % PMA loss per day. The maximum loss, occurring either between the initial scans or at a later time point, differed significantly between survivors (3.66 (IQR 2.03–4.27) % PMA loss per day) and deceased patients (5.48 (IQR 3.09–6.43) % PMA loss per day; *p* = 0.039). Moreover, the maximum muscle decay was significantly higher in male (4.63% PMA per day) compared to female patients (4.40% PMA loss per day; *p* = 0.043). Although higher in the deceased patient group (3.58 (IQR 1.20–4.03) % PMA loss per day) the muscle loss between the first two CTs did not deviate significantly compared to surviving patients (2.22 (IQR 0.77–3.49) % PMA loss per day; *p* = 0.328). No statistically significant differences in muscle decay rates were found between patients with and without ECMO therapy, or patients with and without preexisting conditions (diabetes, arterial hypertension, etc.). Patients that developed an increase in PMA (possibly indicating recovery, *n* = 5) were excluded from the calculations related to muscle loss over the entire time period. Figure 2 depicts the tracking of PMA per CT scan per patient and shows two examples of muscle segmentation during hospitalization.

### Comparison of pandemic waves

During the study period, which encompassed three pandemic waves, the collective was divided into three groups based on data provided by the Robert Koch Institute: wave 1 (03/2020 to 08/2020), wave 2 (08/2020 to 03/2021), and wave 3 (03/2021 to 07/2021) [12]. Group 1 consisted of 15 patients, group 2 comprised 22 patients, and group 3 included 17 patients. In comparison to the other groups, patients admitted during wave 2 exhibited significant differences in terms of mortality, total hospitalization, and ICU days. Survival rates were 87% in group 1, 65% in group 3, and only 27% in wave 2 (*p* = 0.001). Additionally, the average duration of hospitalization (51.23 days, *p* = 0.001) and ICU stay (43.05 days, *p* = 0.005) was significantly shorter for group 2 compared to group 1 (105.9 and 90.80 days, respectively) and group 3 (95.12 and 71.18 days, respectively). Consistent with the clinical data, patients hospitalized during the second wave demonstrated the highest rates of muscle loss. The average daily psoas muscle loss was 2.97% in the first wave, 3.58% in wave 2, and 1.72% in wave 3. The maximum muscle loss was also highest in group 2 (4.81%), compared to group 1 (4.58%) and group 3 (3.94%). Furthermore, there was a nearly significant difference in the daily psoas muscle loss over the entire study period (*p* = 0.053), with group 2 showing a rate of 2.56%, while groups 1 and 3 exhibited rates of 1.47% and 1.42%, respectively. In terms of clinical data, the groups showed differences in the administration of dexamethasone (*p* = 0.001) and tocilizumab (*p* = 0.083), reflecting the shift in therapy strategies over the course of the pandemic. No significant differences between groups were observed for the application and type of ECMO, ventilation parameters (sPEEP, pPeak, RMV, RR, tracheostomy), prone positioning, application of NO therapy, NMBA, or ventilation days. Relevant variables are listed in Table 2.

**Table 2:** Comparison of patient characteristics in the first three COVID waves

Wave	Means			
	1	2	3	*p* value
	*n* = 15	*n* = 22	*n* = 17	
Survival (%)	0.87	0.27	0.65	**0.001**
Hospitalization (days)	105.93	51.23	95.12	**0.001**
ICU stay (days)	90.80	43.05	71.18	**0.005**
sPEEP (cmH_2_O)	16.71	15.73	14.59	0.313
pPeak (cmH_2_O)	30.57	29.73	30.35	0.877
RMV (l/min)	8.32	6.66	7.54	0.248
RR	18.57	18.09	20.41	0.331
Dexamethasone	0.43	0.95	0.82	**0.001**
Tocilizumab	0.00	0.27	0.29	0.084
NMBA	0.43	0.50	0.76	0.127
First loss (%/day)	2.97%	3.58%	1.72%	0.247
Max loss (%/day)	4.58%	4.81%	3.94%	0.740
Overall loss (%/day)	1.47%	2.56%	1.42%	**0.059**

Significant values are printed in bold

### Definition of a discriminatory cut-off and outcome prediction

**Fig. 3:**
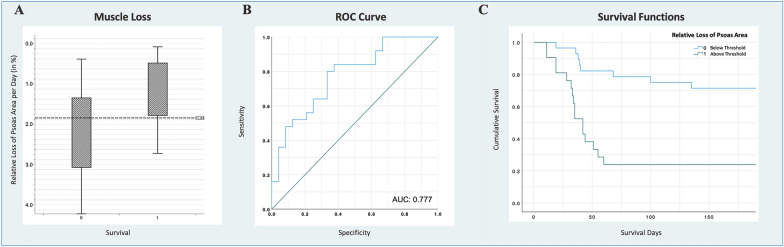
**A** Box plot diagram of relative psoas muscle decay per day in the survivor and non-survivor group. The dotted line represents the defined threshold of 1.84% decline per day. **B**: Receiver operating characteristic (ROC) analysis of the threshold, which proved satisfying discriminatory power with an area undder the curve (AUC) of 0.777. C: Kaplan Meier curves of survival estimates in patients, that did and did not exceed the defined cut-off for muscle decay. Survival was significantly (p < 0.001) reduced in those patients exceeding the threshold

ROC analysis and calculation of the Youden index identified the overall PMA loss of 1.84% per day as cut-off for survival prediction, with good discriminatory power (AUC of 0.777). ROC analysis demonstrated modest discriminative ability for both initial PMA and maximal PMA decline with respect to survival, as evidenced by AUCs of 0.597 and 0.643, respectively. Kaplan–Meier analysis of cumulative survival with patients grouped according to the defined threshold for PMA loss per day revealed significantly (*p* < 0.001) limited survival estimates in patients above the 1.84% cut-off for PMA loss per day, as depicted in Fig. 3.

For the Cox regression analysis for survival and hospital length of stay, variable selection was performed using a backward elimination approach. Muscle loss and initial body BCA parameters were not found to be relevant predictors of length of stay through backward elimination. In contrast, reduced TAMA obtained from the first available scan (*p* = 0.015, OR = 0.981) and the occurrence of high PMA losses between the first two CT scans (*p* = 0.011, OR = 646,339) were significantly associated with survival. Subsequent models were constructed in which the first daily PMA loss was replaced by the maximum and total loss rates, which, due to their nature, are only available at later time points and may have limited clinical relevance. These alternative models demonstrated even higher levels of significance for survival: the *p*-values for the association of survival with maximum and overall loss per day were *p* = 0.004 and *p* < 0.001, respectively. Upon integration of maximum PMA loss per day into the survival prediction model, female gender also reached the significance level (*p* = 0.037). In contrast, variables including RMV, the time interval between admission and intubation (in days), and gender did not show significant associations with survival in all other models. Results of the model incorporating the first measured muscle loss as a covariate are presented in Table 3.

**Table 3:** Cox regression analysis for survival of critically ill patients with severe ARDS due to SARS-CoV-2 infection

	Survival			
	*p* value	Odds ratio		
			Lower	Upper
Gender	0.083	0.393	0.137	1.128
Initial muscle area	**0.015**	0.981	0.965	0.996
Admission to intubation (in days)	0.526	0.976	0.905	1.052
RMV (l/min)	0.395	0.935	0.800	1.092
First PMA loss (%/day)	**0.011**	6.46E+05	20.209	2.07E+10

Significant values are printed in bold

## Discussion

Patients with severe COVID-19 pneumonia were and are a particular challenge to critical care physicians, as they often face prolonged hospital and ICU stays, during which securing positive outcomes is a day-to-day effort. In our study population, mortality was high at 56.6%, and mean hospitalization was 80.2 days (IQR 40.5–103), with an average of 65.2 (IQR 33.25–82.75) days in the ICU. Patients who died had a shorter mean length of stay at 46.13 days (IQR 30.75–54.0), compared to survivors with a mean length of stay of 107.5 days (IQR 71.5–145.75). On average, patients were intubated 4 days after admission, and mechanical ventilation (IMV) was necessary for 56 days (IQR 32.25–68). Extracorporeal membrane oxygenation (ECMO) treatment was performed in 37 (68.5%) patients, of whom 48.65% survived.

 Our study revealed notable disparities in the severity of the three first COVID waves, with particular emphasis on the second wave (08/2020 to 03/2021), during which the majority of patients were admitted to the intensive care unit (ICU) and the mortality rate reached its highest level at 73% in our collective. These findings are in line with the data published by the Robert Koch Institute, which documented the highest utilization of intensive-care capacity and a peak of 6047 deaths attributed to SARS-CoV-2 during the second week of 2021 [12]. At the time, the predominant SARS-CoV-2 variant in Germany was B1.351, also known as the South Africa variant [14]. In our collective, ICU and hospital length of stay were significantly shorter during this wave, compared to waves 1 and 3. As expected, treatment approaches differed over time. Both the antibody tocilizumab, not at all used in wave 1, and dexamethasone were more regularly administered in waves 2 and 3.

In this retrospective study, we investigated long-term skeletal and psoas muscle deterioration during hospitalization of critically ill patients with severe ARDS due to SARS-CoV-2 infection using AI-derived body composition analysis based on routinely performed CT scans. By assessment of tissue proportions at different consecutive time points throughout hospitalization, we revealed significant differences in muscle distribution between age groups, women and men, as well as patients with and without ECMO therapy. Interestingly, the difference in muscle distribution between patients who received ECMO therapy and those who did not, was similar to the physiologically expectable difference in muscle distribution between men and women.

The monitoring of PMA loss demonstrated a non-linear pattern of muscle deterioration, characterized by heterogeneous rates of decline at different time points, while generally exhibiting an overall negative trajectory. Aligned with clinical data, the different rates of muscle wasting between the pandemic waves were highest in the group of patients admitted during the second wave. Among all cases analyzed, 48% exhibited the highest rates of muscle loss between the initial two scans, while 52% experienced the peak decay at later stages. Significant differences in muscle decay rates were observed between survivors and non-survivors throughout the entire monitoring period (first to the last CT scan). Non-survivors experienced a higher rate of 2.62 (IQR 1.4–3.0) % PMA loss per day compared to survivors with a rate of 1.16 (IQR 0.5–1.8) % per day (*p* < 0.001). Additionally, significant disparities were found in the maximum rates of muscle loss between two consecutive assessments, with survivors exhibiting a mean maximal rate of 3.66 (IQR 2.03–4.27) % per day and decedents demonstrating a higher rate of 5.48 (IQR 3.09–6.43) % per day (*p* = 0.039). The initial PMA loss, occurring between the first two scans and presumably most relevant for timely intervention, did not exhibit significant differences between survival and non-survival groups. However, it demonstrated a strong association with survival in Cox regression analysis (*p* = 0.011), alongside the initial TAMA (*p* = 0.015). Furthermore, a robust long-term threshold of 1.84% PMA loss per day was identified for survival prediction, demonstrating considerable discriminatory power (AUC 0.777).

Previous studies have underlined the rapid and significant muscle decay in patients after ICU admission and demonstrated the potential of muscle monitoring in critically ill patients to identify individuals who face adverse outcomes during and after hospitalization [15–17]. Our study stands out from other investigations in three respects: the time period by which patients were observed, the time intervals between muscle measurements, and the modality of monitoring. While previous studies were mainly based on daily measurements over the first 7 to 10 days, our observations were intermittent, but covered almost the entire hospital stay—which seems reasonable as some critically ill patients, like those with SARS-CoV-2 infection, often require much longer ICU stays [18]. Instead of following predetermined intervals between monitoring time points, our approach involved follow-up CT scans that were conducted in response to clinical indications. Interestingly, our method revealed an average PMA loss rate of 1.88% per day, which is exactly in the range of previously published muscle atrophy rates of 21.8% and 17.7% over 10 days. Moreover, the high relative loss of PMA per day (2.62%) in the deceased group underlines the correlation of muscle wasting with the severity of the underlying condition [4].

While previously deployed BIA and US profit from ease of availability at the bedside, both are dependent on availability of medical personnel and have limitations regarding reproducibility and susceptibility to error. BIA measurements can be distorted due to fluid accumulation in soft tissue, while US is highly examiner-dependent [3, 19, 20]. In contrast, application of image segmentation tools allows for more objective and reproducible measurements of not only one, but multiple tissue components (SAT, VAT, TAMA, and PMA). As CT derived BCA is applicable not only to in-house scans, but also to externally acquired images, retrospective long-term analysis of muscle atrophy becomes feasible even in patients who are transferred to specialized centers during the course of their disease. In addition to radiation exposure, the availability of CT scans is a clear limitation for the application of sufficient monitoring [6]. This constraint has been relativized with the outbreak of the COVID-19 pandemic and the associated recommendation of international guidelines to perform CT imaging for the (repeated) assessment of patients with SARS-CoV-2 infections [8]. Our data show that BCA derived from unscheduled CT scans can serve as a viable monitoring tool providing relevant information about patient’s physical condition at admission and its deterioration during hospitalization. The desirable implementation of our method into routine practice would save additional BIA and US examinations, thereby relieving staff capacity, and has several potential clinical applications.

A major concern during the pandemic was the limited availability of extracorporeal membrane oxygenators and the resulting need for selecting patients most likely to benefit from it [21, 22].

Decisions regarding ECMO therapy often needed to be made on an individual basis, as there was no appropriate blanket approach to COVID-19 patients [23]. As our data suggest, additional BCA-derived knowledge about a patient’s current physical status, the muscle loss already incurred and the associated prognosis might therefore be a useful contribution to decision-making. During the pandemic, survival prediction of COVID-19 patients was complicated, particularly because conventional scores, such as the SOFA (sequential organ failure assessment) score showed limited applicability. Today, we know that factors such as age and BMI have a relevant impact [24, 25]. However, BMI is a notoriously inaccurate score, as it ignores the relative proportions of different tissue types that contribute to a person’s total weight. More accurate predictions might be made by application of tissue segmentation, as indicated by our results. In this context, future studies on patient’s fat tissue distribution might be of special interest, as SARS-CoV-2 infection of adipose tissue seems to contribute to the severity of COVID-19 [26]. Another promising area of application concerns patients who have overcome the critical phase of their disease. Most patients who survive critical illness have mid- to long-term cognitive, psychological, and/or physical impairments, which are collectively referred to as post-intensive care syndrome (PICS) [27, 28]. Intensive care acquired weakness (ICUAW), marked by severe muscle wasting during ICU stays, is one column of PICS. It represents a neuromuscular dysfunction that affects peripheral as well as respiratory muscles and adversely alters short- and long-term outcomes [1, 29, 30]. Recent studies underline the considerable impact of ICUAW on quality of life after hospital discharge [2, 31]. As no effective treatment has yet been found [32], prevention plays a key role making early identification of patients at risk crucial [1]. Even though muscle deterioration is not pathognomonic for ICUAW [33], we could show that BCA is a viable tool to identify patients with severe muscle decay, whose muscle force should be evaluated and who should receive preventive support accordingly.

### Limitations

Due to the retrospective study design, selection bias is unavoidable. Although the methodology is robust and worked particularly well in our collective, detecting minor and major changes of muscle area, it is important to note that less severely ill patients, who might yield less conclusive results, are underrepresented. The retrospective design makes it difficult to draw conclusions about causality of muscle loss. This hinders the evaluation of factors known to influence muscle wasting, such as administration of NMBAs or application and composition of parenteral nutrition [34]. Moreover, the impact of clinical data is constrained by the varying admission time points, which may lead to an underestimation of their significance. For instance, ventilatory data from patients transferred to our facility already requiring ECMO cannot be directly compared to data obtained prior to intubation. In addition, the generalizability and validity of our findings are limited by the moderate sample size. However, the highly significant differences between survivors and deceased patients already observed in our moderately large patient population suggest the validity of our results and underscore the need for image segmentation in routine clinical practice. This would improve our understanding of the relationship between individual tissue loss and clinical parameters and could help us in developing even more accurate prognostic markers.

## Conclusion

Critically ill COVID-19 patients suffer severe muscle wasting and the extent of muscle loss correlates with their survival. Intermittent BCA derived from clinically indicated CT scans provides a monitoring tool, which enables identification of individuals at risk for adverse outcomes and has great potential to aid decision-making in critical care.

## Data Availability

The datasets generated during and analyzed during the current study are available from the corresponding author on reasonable request.

## Abbreviations

AI: Artificial intelligence
ANOVA: Analysis of variance
ARDS: Acute respiratory distress syndrome
BCA: Body composition analysis
BIA: Bioelectrical impendence analysis
BMI: Body mass index
CT: Computed tomography
ECMO: Extracorporeal membrane oxygenation
ICU: Intensive care unit
IMV: Invasive mechanical ventilation
NMBA: Neuromuscular blocking agents
PACS: Picture archiving and communication system
PICS: Post-intensive care syndrome
PMA: Psoas muscle area
pPeak: Peak inspiratory pressure
RMV: Respiratory minute volume
RR: Respiratory rate
SAT: Subcutaneous adipose tissue
SMA: Skeletal muscle area
SOFA: Sequential Organ Failure Assessment
sPEEP: Set positive end-expiratory pressure
TAMA: Total abdominal muscle area
US: Ultrasound
VAT: Visceral adipose tissue

## References

[1] Vanhorebeek I, Latronico N, Van den Berghe G (2020). ICU-acquired weakness. Intensive Care Med.

[2] Herridge MS (2011). Functional disability 5 years after acute respiratory distress syndrome. N Engl J Med.

[3] Nakanishi N (2019). Monitoring of muscle mass in critically ill patients: comparison of ultrasound and two bioelectrical impedance analysis devices. J Intensive Care.

[4] Puthucheary ZA (2013). Acute skeletal muscle wasting in critical illness. JAMA.

[5] Weijs PJ (2014). Low skeletal muscle area is a risk factor for mortality in mechanically ventilated critically ill patients. Crit Care.

[6] Looijaard W, Molinger J, Weijs PJM (2018). Measuring and monitoring lean body mass in critical illness. Curr Opin Crit Care.

[7] Beetz NL (2021). First PACS-integrated, artificial intelligence-based software tool for rapid and fully automatic analysis of body composition from CT in clinical routine. JCSM Clin Rep.

[8] Rubin GD (2020). The role of chest imaging in patient management during the COVID-19 pandemic: a multinational consensus statement from the Fleischner Society. Chest.

[9] Beetz NL (2021). Artificial intelligence-based analysis of body composition in Marfan: skeletal muscle density and psoas muscle index predict aortic enlargement. J Cachexia Sarcopenia Muscle.

[10] Fehrenbach U (2021). CT body composition of sarcopenia and sarcopenic obesity: predictors of postoperative complications and survival in patients with locally advanced esophageal adenocarcinoma. Cancers (Basel).

[11] Kim D (2019). Comparative assessment of skeletal muscle mass using computerized tomography and bioelectrical impedance analysis in critically ill patients. Clin Nutr.

[12] Robert-Koch-Institiut. Pandemieradar - Der Pandemieradar des Robert-Koch-Instituts zeigt täglich aktualisierte Daten zum Infektionsgeschehen. 2023. https://www.rki.de/DE/Content/InfAZ/N/Neuartiges_Coronavirus/Situationsberichte/COVID-19-Trends/COVID-19-Trends.html?__blob=publicationFile#/home. Accessed 14 May 2023.

[13] Force ADT (2012). Acute respiratory distress syndrome: the Berlin Definition. JAMA.

[14] Robert-Koch-Institiut. Bericht zu Virusvarianten von SARS-CoV-2 in Deutschland. 2021.

[15] Mayer KP (2020). Acute skeletal muscle wasting and dysfunction predict physical disability at hospital discharge in patients with critical illness. Crit Care.

[16] Tanaka K, Yamada T (2021). Ultrasound measurement of septic shock-induced acute skeletal muscle atrophy in intensive care unit. PM R.

[17] Schefold JC, Bierbrauer J, Weber-Carstens S (2010). Intensive care unit-acquired weakness (ICUAW) and muscle wasting in critically ill patients with severe sepsis and septic shock. J Cachexia Sarcopenia Muscle.

[18] Nguyen NT (2021). Outcomes and mortality among adults hospitalized with COVID-19 at US medical centers. JAMA Netw Open.

[19] Hides J (1995). Ultrasound imaging in rehabilitation. Aust J Physiother.

[20] Bunnell A (2015). Quantitative neuromuscular ultrasound in intensive care unit-acquired weakness: a systematic review. Muscle Nerve.

[21] Phua J (2020). Intensive care management of coronavirus disease 2019 (COVID-19): challenges and recommendations. Lancet Respir Med.

[22] Heinsar S, Peek GJ, Fraser JF (2020). ECMO during the COVID-19 pandemic: when is it justified?. Crit Care.

[23] Camporota L (2020). Identification of pathophysiological patterns for triage and respiratory support in COVID-19. Lancet Respir Med.

[24] Raschke RA (2021). Discriminant accuracy of the SOFA score for determining the probable mortality of patients with COVID-19 pneumonia requiring mechanical ventilation. JAMA.

[25] Gao M (2021). Associations between body-mass index and COVID-19 severity in 6.9 million people in England: a prospective, community-based, cohort study. Lancet Diabetes Endocrinol.

[26] Martinez-Colon GJ (2022). SARS-CoV-2 infection drives an inflammatory response in human adipose tissue through infection of adipocytes and macrophages. Sci Transl Med.

[27] Inoue S (2019). Post-intensive care syndrome: its pathophysiology, prevention, and future directions. Acute Med Surg.

[28] Adrion C (2020). Enhanced Recovery after Intensive Care (ERIC): study protocol for a German stepped wedge cluster randomised controlled trial to evaluate the effectiveness of a critical care telehealth program on process quality and functional outcomes. BMJ Open.

[29] Hermans G, Van den Berghe G (2015). Clinical review: intensive care unit acquired weakness. Crit Care.

[30] Iwashyna TJ (2010). Long-term cognitive impairment and functional disability among survivors of severe sepsis. JAMA.

[31] Van Aerde N (2020). Five-year impact of ICU-acquired neuromuscular complications: a prospective, observational study. Intensive Care Med.

[32] Friedrich O (2015). The sick and the weak: neuropathies/myopathies in the critically ill. Physiol Rev.

[33] Callahan LA, Supinski GS (2009). Sepsis-induced myopathy. Crit Care Med.

[34] Farhan H (2016). Acquired muscle weakness in the surgical intensive care unit: nosology, epidemiology, diagnosis, and prevention. Anesthesiology.

